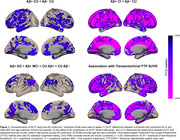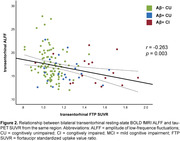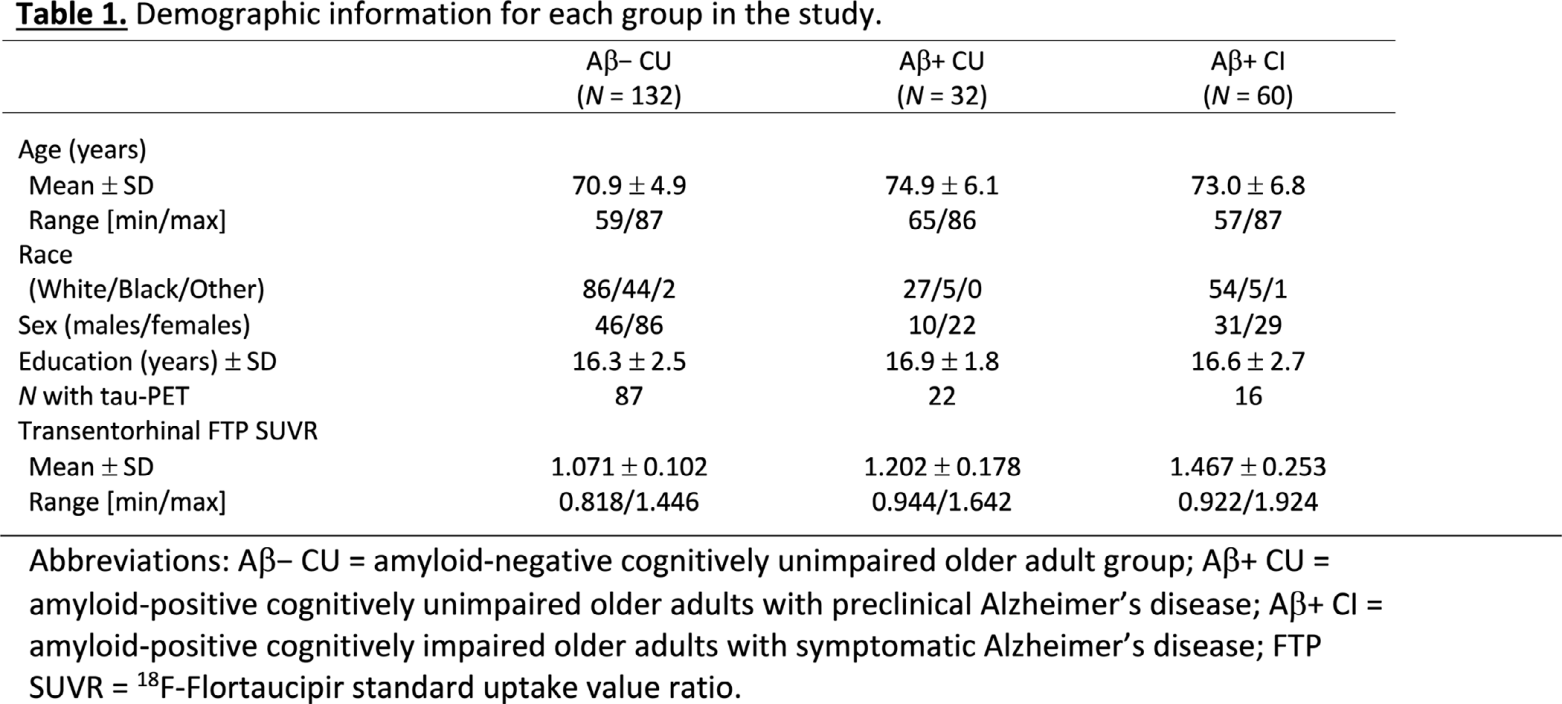# BOLD fMRI signal changes in preclinical Alzheimer’s disease

**DOI:** 10.1002/alz.093549

**Published:** 2025-01-09

**Authors:** Stanislau Hrybouski, Sandhitsu R. Das, Long Xie, Xueying Lyu, Laura E.M. Wisse, Melissa Kelley, Jacqueline Lane, Monica Sherin, Michael DiCalogero, Ilya M. Nasrallah, John A. Detre, Paul A. Yushkevich, David A Wolk

**Affiliations:** ^1^ University of Pennsylvania, Philadelphia, PA USA; ^2^ Penn Image Computing and Science Laboratory (PICSL), University of Pennsylvania, Philadelphia, PA USA; ^3^ Penn Alzheimer’s Disease Research Center, University of Pennsylvania, Philadelphia, PA USA; ^4^ Lund University, Lund Sweden

## Abstract

**Background:**

Previous work suggests functional abnormalities in the human brain in preclinical Alzheimer’s disease. However, little has been explored about the relationship between BOLD fMRI signal amplitude/energy over time and AD pathology. In this work we analyzed the effects of AD progression on amplitude of low‐frequency fluctuations (ALFF) during resting‐state fMRI scans both at the whole‐brain level and at a more granular level, focused on regions of the medial temporal lobe (MTL) that are most vulnerable to AD pathology.

**Method:**

In this cross‐sectional study, we analyzed data from 224 individuals from the Penn ADRC cohort (Table 1). All participants underwent structural and functional MRI on a Siemens 3T Prisma system, and ^18^F‐Florbetaben or ^18^F‐Florbetapir amyloid‐PET imaging. 125 participants also underwent ^18^F‐Flortaucipir tau‐PET scans. Functional images were preprocessed using a custom implementation of *fMRIprep* and ALFF was extracted using Conn software. In the whole‐brain analyses we performed voxelwise GLMs with age and sex as covariates.

**Results:**

We observed reduced ALFF in both preclinical AD (Amyloid‐positive (Aβ+) cognitively unimpaired, CU) and Aβ+ cognitively impaired (CI) individuals. Relative to Aβ‐ controls, individuals with preclinical AD displayed lower ALFF in frontal, parietal and temporal association cortices (Figure 1, top left). CI individuals displayed lower ALFF in most of the brain, except in inferior temporal cortex, temporal pole, and MTL. The effect of AD progression on ALFF was characterized by a progressive reduction primarily in frontal and parietal regions that roughly align with the anatomy of the default mode network. In contrast, transentorhinal tau pathology was negatively associated with ALFF in frontal and anterior temporal lobes, as well as insula and MTL (Figure 1 bottom right). Negative association between tau burden and MTL ALFF was observed in all main MTL subregions, and strongest in the transetorhinal cortex (Figure 2).

**Conclusion:**

We conclude that: (1) ALFF might be a promising biomarker for studying functional abnormalities in preclinical AD, and (2) based on the spatial topography of amyloid and tau effects on ALFF (Figure 1 bottom), there is likely a differential effect of of the two on ALFF that needs to be further explored.